# Role of forensic odontology in the identification of victims of major mass disasters across the world: A systematic review

**DOI:** 10.1371/journal.pone.0199791

**Published:** 2018-06-28

**Authors:** Ghevaram Prajapati, Sachin C. Sarode, Gargi S. Sarode, Pankaj Shelke, Kamran H. Awan, Shankargouda Patil

**Affiliations:** 1 Department of Oral Pathology and Microbiology, Dr. D.Y. Patil Dental College and Hospital, Dr. D.Y. Patil Vidyapeeth, Pune, India; 2 College of Dental Medicine, Roseman University of Health Sciences, South Jordan, Utah, United States of America; 3 Department of Maxillofacial Surgery and Diagnostic Sciences, Division of Oral Pathology, College of Dentistry, Jazan University, Jazan, Saudi Arabia; Navodaya Dental College and Hospital, INDIA

## Abstract

**Background:**

Forensic odontology (FO) is regarded in the literature as one of the most reliable and economical scientific methods for victim identification in mass disasters (MDs). The present paper systematically reviews the role of forensic odontologists in various global MDs.

**Method:**

A comprehensive search of the literature databases (PubMed, Medline, SCOPUS, Web of Science and Google Scholar), along with cross-referencing published peer-reviewed articles, was conducted. The search included full texts, abstracts or titles, had no inclusion year limit (searched until September 2017) and was limited to the English language. Keywords included a combination of ‘Forensic odontology’, ‘Dental records’, ‘Victim identification’, ‘Natural mass disaster’, ‘Criminal mass disaster’, ‘Accidental mass disaster’ and ‘Victim disaster’.

**Results:**

Of the included disasters (20), 12 (57.14%) were accidental, 5 (23.80%) natural and 3 (19.04%) were criminal. The maximum number of victims was associated with the Japan tsunami (15892), followed by the Thailand tsunami (4280) and the Estonia ferry disaster (852). A total of 23654 victims were reported, of which 20569 (86.96%) were positively identified. Reports from 17 MDs included the use of FO in victim identification [3025 (14.70%) cases]. In addition, 1094 victims (5.31%; from 7 papers) were identified using FO in combination with other methodologies. The highest percentage of victims was identified using FO following the Kentucky air crash (47; 100%), followed by the Newark air crash (38; 76%), the Nepal air crash (10; 71.42%), the France air crash (56; 65.88%), the Australian bushfire (14; 63.63%), and the Estonia ferry disaster (57; 60.63%).

**Conclusion:**

FO has played a significant role in victim identification in several MDs around the world. The success of FO-based identification is heavily dependent on the availability of ante-mortem records from general dental practitioners. Hence, adequate knowledge about FO and appropriate dental record keeping among general dental practitioners are critical.

## Introduction

Mass disasters (MDs) are sudden, violent, unexpected and indiscriminate events that are usually associated with a large number of casualties, and they require significant resources for management. MDs are broadly categorized as natural, accidental or criminal [1]. The Centre for Research on the Epidemiology of Disasters defines a disaster as “a situation or event which overwhelms local capacity, necessitating a request to a national or international level for external assistance; an unforeseen and often sudden event that causes great damage, destruction and human suffering”. For a disaster to be entered into the database, at least one of the following criteria must be fulfilled: 10 or more people reported killed; 100 or more people reported affected; declaration of a state of emergency; or a call for international assistance [2]. Disasters can also be further divided into open disasters, closed disasters or open and closed disasters. Disasters such as earthquakes, tsunamis, and train accidents belong to the open MD category. In these disasters, the names of the victims are usually unknown. On the other hand, air crashes, ferry disasters, and hotel fires are examples of closed disasters, where the names of the victims can usually be obtained [3]. In recent times, the incidences of MDs and associated casualties have increased many-fold due to the expansion of travel facilities, a greater variety of transport types, an increase in terrorism, and an increase in unusual climatic conditions, among other reasons. Therefore, the need for additional resources and development so that disaster management teams can function effectively has increased. An essential aspect of disaster management is the identification of post-mortem remains, which is usually carried out by forensic experts. Forensic identification of the victims of these MDs is essential, not only for humanitarian reasons but also for civil or criminal investigative needs. It is a very challenging task because dead bodies are often mutilated to such an extent that they cannot be identified by general physical examination alone [3]. In such situations, forensic anthropology, fingerprint analysis, forensic odontology (FO), radiology and DNA typing can be used for victim identification.

Forensic dentistry, which is also referred to as FO, is an area of dentistry concerned with the correct management, examination, evaluation, and presentation of dental evidence in criminal or civil legal proceedings in the interest of justice [4]. Various methods employed in FO for identification include review of dental case records, anthropological assessments, and analyses of, restorations, dentures, radiographs, bite marks and intra-oral photographs, as well as, cheiloscopy and rugoscopy. Because of the protective environment inside the oral cavity, dental pulp is considered as most reliable source for DNA-based identification procedures. Forensic odontologists are not only trained to solve individual cases but are also capable of handling victim identification in MDs [5]. FO is regarded in the literature as one of the most reliable and economical scientific methods for victim identification in MDs. However, to confirm this fact, there is a need for a systematic analysis of the role of forensic odontologists in natural, criminal and accidental MDs. Therefore, the aim of the present study was to systematically review the role of FO in the identification of victims following MDs.

## Materials and methods

### Focused question

What is the role of FO in the identification of victims of various MDs across the world?

### Database sources

This systematic review was conducted according to the guidelines of the preferred reporting items for systematic reviews and meta-analyses. (S1 Table). A comprehensive search of the literature databases (PubMed, Medline, SCOPUS, Web of Science and Google Scholar), along with cross-referencing of published peer-reviewed articles, was conducted. The search included full-text articles, abstracts or titles, had no inclusion year limit (searched until September 2017 for published peer-reviewed papers) and was limited to the English language.

### Search strategy

Keywords included a combination of ‘Forensic odontology’, ‘Dental records’, ‘Victim identification’, ‘Natural mass disaster’, ‘Criminal mass disaster’, ‘Accidental mass disaster’ and ‘Victim disaster’. To identify relevant records, keywords were connected with the Boolean operator ‘AND’. Thus, twelve search sub-strings were utilized to identify the relevant articles. Details of the output of search strings from the PUBMED and SCOPUS databases are shown in Table 1. Citations from selected references and bibliographic links taken from the studies were included in this review. Journals related to subjects such as FO, forensic medicine, oral pathology and oral medicine were also searched using the keywords above. In addition, when a relevant title with/without an abstract was identified and the full-text article was not available in the electronic databases, the full-text was obtained from the grey literature.

**Table 1:** Summary of the results of the PubMed and scopus search strategy.

Sr. No	Search strategy	PubMed		Scopus	
		Hits	Selected	Hits	Selected
1	Forensic odontology AND natural mass disaster	7	0	23	3
2	Forensic odontology AND criminal mass disaster	11	0	18	1
3	Forensic odontology AND accidental mass disaster	0	0	2	0
4	Dental records AND natural mass disaster	6	1	16	2
5	Dental records AND criminal mass disaster	0	0	13	0
6	Dental records AND accidental mass disaster	0	0	1	0
7	Victim identification AND criminal mass disaster	9	2	31	2
8	Victim identification AND natural mass disaster	11	4	58	13
9	Victim identification AND accidental mass disaster	2	0	6	0
10	Forensic odontology AND Disaster Victim	55	15	137	38
11	Forensic odontology AND Victim, Disaster	55	13	137	30
12	Forensic odontology AND Victims, Disaster	36	12	137	29

### Eligibility criteria

Inclusion criteria were as follows: Peer-reviewed, published papers written in English that reported victim identification in a MD were included in the systematic review. The PICO strategy was used to address articles as follows: involved participants (P)–any age or sex; intervention (I)–forensic identification of victims; control (C)–not applicable; and outcome (O)–forensic odontological identification.

The following articles were excluded: a) Narrative reviews, critical reviews, commentaries, opinions and editorials; b) articles on MDs that did not mention the modes of victim identification; and c) papers published in any language other than English.

### Data extraction and quality assessment

Two authors conducted the literature searches (GP and GSS), study selection (GP and SCS), and data extraction (GP and SCS) independently. The extracted data included authors, year of publication, year of disaster, study size, disaster type, disaster subtype, country, state, total number of victims, total number of victims identified, total number of victims identified by FO, whether identification by FO was used in combination with other methods and other methods of identification. Disagreement was resolved by discussion and consensus reached through a third party (PS).

Study quality was determined by the primary data extractors (GP and SCS) and confirmed by at least one other co-author. To inform the assessment of study quality, we considered factors in the National Institutes of Health, Quality Assessment Tool for Observational Cohort and Cross-Sectional Studies. [6] While assessing the quality of articles, the aforementioned data extraction items were kept in mind. The quality of each included article was categorized to good, fair or poor.

## Results

### Literature search

**Fig 1:**
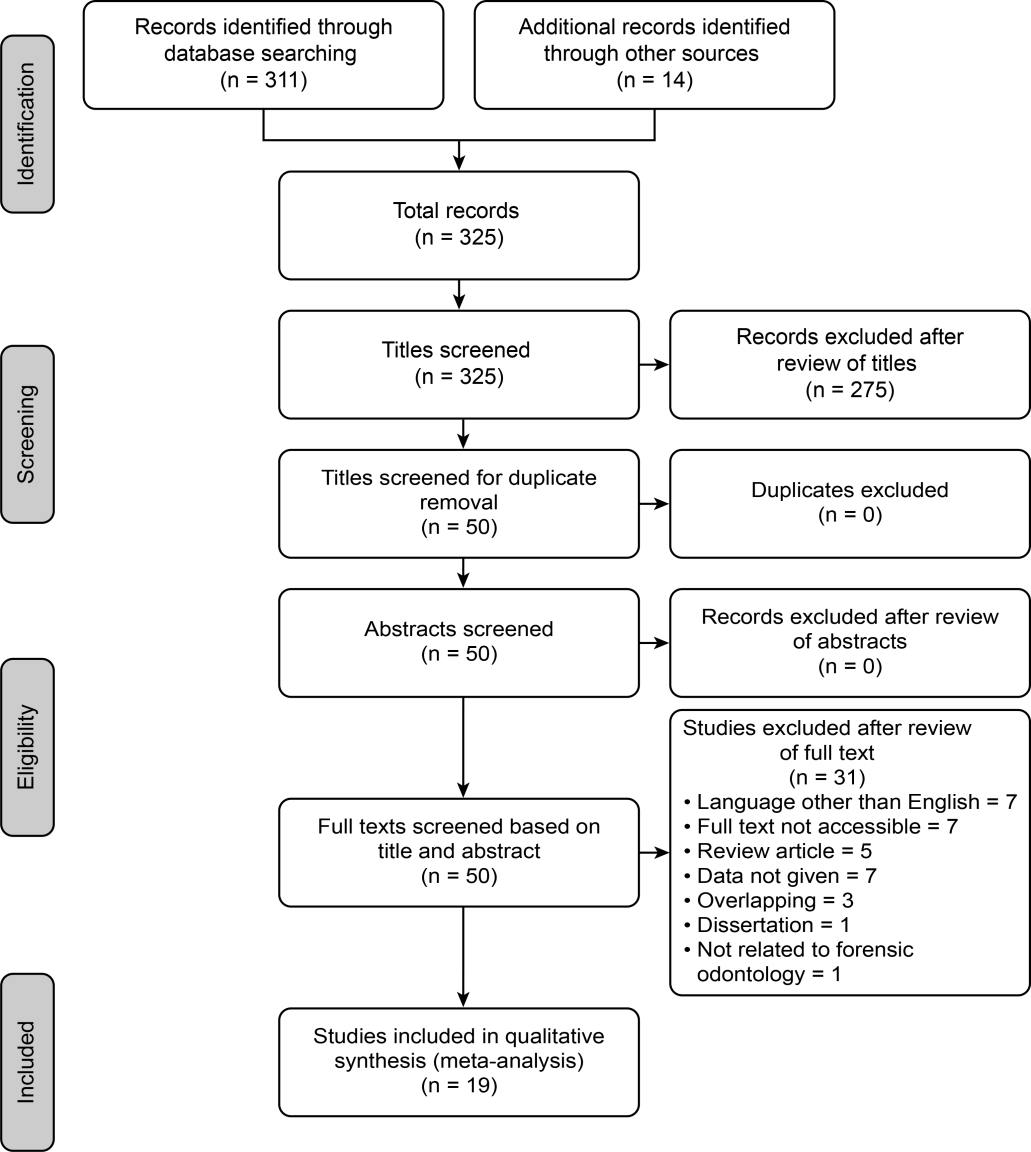
PRISMA flow diagram.

Fig 1 summarizes the details of the entire selection process for article retrieval by the aforementioned search approaches. The database searches revealed 47 articles in PubMed, 118 in Scopus, 146 in Google Scholar and 14 from other sources (cross-references, specialty journal searches). The summary of the PubMed and Scopus searches is presented in Table 2. In summation, after the removal of common references from the databases, a total of 325 records were identified; 275 records were excluded after review of the titles. None of the remaining articles (50) were removed after screening for duplicate publications and initial review of the abstracts. In total, 31 articles were excluded after review of the full text because of the following reasons: published in a language other than English (7), the full text was not accessible (7), the publication was a review article (5), the data were not mentioned (07), overlapping data (3), a dissertation (1) and FO methods were not applied (1). Ultimately, 19 articles (20 MDs) that met the inclusion criteria were selected for the study. The summary of the victim identification methods used in these 20 MDs [7–25] is shown in Table 2 and the details of each MD are presented in Table 3.

**Table 2:** Summary of victim identification methods used in mass disasters.

Parameters		Number (%)
Total number of mass disasters		20
Total number of victims		23654
Total number of identifications		20569 (86.95)
Forensic odontology (FO)		3025 (14.70)
FO + others		1094 (5.31)
Methods other than FO		16447 (79.96)
	DNA	241 (1.17)
	Fingerprint	1250 (6.07)
	Personal	98 (0.47)
	Combination	178 (0.86)
	Physical	6 (0.02)
	Visual	14029 (68.20)
	Other	129 (0.622)
	Fingerprint + DNA	9 (0.04)
	Fingerprint + Physical	119 (0.57)
	Fingerprint + Physical + DNA	12 (0.058)
	Physical + DNA	384 (1.86)
	Hip implant	1 (0.004)
	NA	6 (0.02)

**Table 3:** Details of victim identification in individual mass disasters.

Author	Year	Disaster	Disaster subtype	Country	State/city	Total number of victims	Number of identified victims (%)	Number identified by FO (%)	FO used in combination with other methods	Other methodologies used for identification
Bastiaan et al. [7]	1984	Natural	Bushfires	Australia	Victoria	72	22 (30.55)	14 (63.63)	NA	8
Solheim et al. [8]	1992	Accident	Ferry	Norway	Oslo	158	158 (100)	107 (67.72)	NA	NA
Brkic et al. [9]	1997	Criminal	War	Croatia	Petrinja	46	27 (58.69)	7 (25.92)	NA	Personal, 20
Brkic et al. [10]	2000	Criminal	War	Croatia	Slavonia	1000	824 (82.4)	206 (25)	527	Others, 91
Soomer et al. [11]	2001	Accident	Ferry	Europe	Estonia	852	94 (11.03)	57 (60.63)	NA	NA
Dumanèiæ et al. [12]	2002	Accident	Train	Croatia	Zagreb	152	111 (73.02)	0	6	Fingerprint, 20; Personal, 42; Combination, 49
	2002	Accident	Air crash	Croatia	Vrbovec	176	166 (94.31)	0	37	Fingerprint, 6; Personal, 58; Combination, 102
Hutt et al. [13]	2005	Accident	Air crash	France	Strasbourg	87	85 (97.7)	56 (65.88)	NA	Other, 29
Bux et al. [14]	2006	Accident	Air crash	Nepal	Jomsom	18	14 (77.77)	10 (71.42)	FO+Anthropological+Personal, 4	NA
Schuller-Götzburg et al. [15]	2007	Natural	Tsunami	Thailand	Phuket	4280	2679 (62.59)	1105 (41.24)	FO+Personal, 206; FO+Fingerprint, 49; FO+Fingerprint+Personal, 64; FO+DNA, 1; FO+Fingerprint+DNA, 1; FO+Fingerprint+Personal+DNA, 3; FO+Personal+DNA, 22	Fingerprint, 670; Fingerprint+DNA, 9; Fingerprint+Personal, 119; Fingerprint+Personal+DNA, 12; DNA, 27; Personal, 6; Personal+DNA, 384; Other, 1
Tan et al. [16]	2007	Accident	Air crash	Indonesia	Southern Sumatra	104	6 (5.7)	2 (33.33)	NA	Fingerprint, 2; Age, 1; Personal, 1
Prieto et al. [17]	2007	Criminal	Terrorist attacks	Spain	Madrid	191	191 (100)	0	NA	DNA, 31; Fingerprint, 145; Combination, 15
Hinchliffe et al. [18]	2009	Natural	Bushfires	Australian	Victoria	173	172 (99.42)	69 (40.11)	NA	NA
Trengrove et al. [19]	2011	Natural	Earthquake	New Zealand	Christchurch	181	177 (97.79)	58 (32.76)	FO+Fingerprint+DNA, 25	DNA, 7; Fingerprint, 76 Physical/visual, 11
Hinchliffe et al. [20]	2011	Accident	Air crash	USA	Kentucky	50	47 (94)	47 (100)	NA	NA
Bush et al. [21]	2011	Accident	Air crash	USA	Newark	50	50 (100)	38 (76)	NA	Combination, 12
Manhart et al. [22]	2012	Accident	Vehicle	Germany	Autobahn A19	8	8 (100)	0	0	DNA, 7; Hip implant, 1
Barberia et al. [23]	2015	Accident	Train	Barcelona	Castelldefels Platja station	12	12 (100)	0	0	DNA, 12; Fingerprint, 11
Obafunwa et al. [24]	2015	Accident	Air crash	Nigeria	Lagos	152	148 (97.36)	15 (10.13)	DNA+FO, 133	NA
Iino et al. [25]	2015	Natural	Tsunami	Japan	East Japan	15892	15736 (99.01)	1259 (8)	NA	DNA, 157; Fingerprint, 315; Personal, 14005

#### Disaster type and geographic location

Of the included disasters (20), **12 (57.14%)** were accidental [8,11–14,16,20–24], **5 (23.80%)** were natural [6,15,18,19,25] and **3 (19.04%)** were criminal [9,10,17]. The twelve accidental MDs included airplane crashes [12–15,16,20,21,24] (n = 7; 23.80%), ferry accidents [8,11] (n = 2; 9.52%), train accidents [12,23] (n = 2; 9.52%) and a vehicle accident [22] (n = 1; 4.76%). Of the 5 natural MDs, 2 (9.52%) were bushfires [5,16], 2 (9.52%) were tsunamis [15,25] and 1 (4.76%) was an earthquake [19]. Ten MDs (52.38%) were in Europe (Croatia [9,10,12], 4; Estonia [11], 1; France [13], 1; Germany [22], 1; Spain [17,23], 2; and Norway [8],1), 4 (19.04%) were in Asia (one each in Nepal [14], Thailand [15], Indonesia [16] and Japan [25]), 2 (9.52%) were in Australia [7,18], 2 (9.52%) were in the USA [20,21] and 1 (4.76%) each was in Africa (Nigeria) [24] and New Zealand [19].

#### Victim identification

Of the included MDs, the greatest number of victims was associated with the Japan tsunami [25] (15892), followed by the Thailand tsunami [15] (4280) and the Estonia ferry disaster [11] (852). The Germany vehicle accident [22] (8), the Barcelona train accident [23] (12) and the Nepal airplane crash [14] (18) were associated with the lowest numbers of victims. The details of the victim identification for the Norway ferry disaster were not available [8]. A total of 23654 victims were reported from 20 MDs, of which 20569 victims (86.95%) were positively identified. All victims (100%) were identified in the Spain terrorist attack [15], the Newark air crash [21], the Barcelona train accident [23] and the Germany vehicle accident [22]. The greatest number of victims was identified from the Australian bushfire of 2009 [18] (172; 99.42%), followed by the Japan tsunami [25] (15736; 99.01%), the New Zealand earthquake [19] (177; 97.79%), the France air crash [13] (85; 97.7%), the Nigeria air crash [24] (148; 97.36%) and the Croatia air crash [12] (166; 94.31%). In contrast, lower identification rates were observed in the Indonesia air crash [16] (6; 5.7%), the Europe ferry disaster [11] (94; 11.03%) and the Australian bushfire of 1984 [7] (22; 30.55%).

#### Mode of victim identification

Of 23654 victims, 20569 (86.95%) were positively identified using various forensic methodologies. Of the 20569 identified victims, 16447 (79.96%) were identified using methodologies other than FO. Among these methodologies, the most commonly used were visual/personal identification [14029 (68.2%)], fingerprinting [1250 (6.07%)] and DNA identification [241 (1.17%)]. Details for the other methods are given in Table 2. In two accidental MDs (Germany vehicle accident [22] and Barcelona train accident [23]) and in one criminal MD (Spain terrorist attack [17]), FO was not used for victim identification. Of the 20569 (86.95%) victims identified, FO was used for the identification of 3025 victims (14.70%). In addition, 1094 victims (5.31%; from 7 papers [9,11,13,14,23]) were identified using FO in combination with other methodologies (Table 2).

#### Forensic odontological identification

Of the 20 MDs, 17 involved the use of FO for victim identification. [7–16,18,19–21,24,25] All victims were identified using FO in the Kentucky air crash (47; 100%) [20]. The highest percentage of victims was identified using FO was in the Newark air crash [21] (n = 38; 76%), followed by the Nepal air crash [14] (n = 10; 71.42%), the France air crash [13] (n = 56; 65.88%), the Australian bushfire [7] (n = 14; 63.63%), and the Estonia ferry disaster [11] (n = 57; 60.63%). In contrast, lower percentages of victim identification were observed following the Japan tsunami [25] (n = 1259; 8%) and the Nigeria air crash [24] (n = 15; 10.13%). In total, 1094 (n = 5.31%) victims (from 7 papers [10,12,14,15,19,24]) were identified using FO in combination with other methodologies (Table 2). In the Croatia train and air crash accidents [12], FO was exclusively used in combination with other identification methods for the identification of the victims (n = 6 and n = 37, respectively). Details on the other methodologies used in combination with FO were not available for the Croatia criminal attack [10], train accident [12] and air crash [12] MDs. In the Spain terrorist attack [17] (191 victims), the Barcelona train accident [23] (12 victims) and the Germany vehicle accident [22] (8 victims), all the victims were identified using a methodology other than FO.

## Discussion

Among the three types of MDs (natural, criminal and accidental), natural MDs are most commonly characterized by a large number of victims [5]. The five natural MDs in this review include bushfires [7,18], tsunamis [15,25] (Thailand and Japan), and earthquakes [19] (New Zealand). The greatest numbers of victims were associated with the Japan tsunami [25] (n = 15892), followed by the Thailand tsunami [15] (n = 4280) and the New Zealand earthquake [19] (n = 181). Victims in such situations can be scattered over large areas that extend for miles. Moreover, victims who are transients, homeless individuals or tourists pose problems for identification due to unavailability of ante-mortem records. Despite these issues, significant numbers of victims were identified in the Australian bushfire of 2009 [18] (n = 172; 99.42%), the Japan tsunami [25] (n = 15736; 99.01%) and the Thailand tsunami [15] (n = 2679; 62.59%). Forensic odontologists may face unique problems due to compromised infrastructure, destruction of ante-mortem records from local dental clinics, and loss of communication lines. All these factors can delay or preclude the prompt identification of victims, which is strongly reflected in low rates of forensic odontological identification in the Japan tsunami [25] (n = 1271; 8.07%) and the New Zealand earthquake [19] (n = 33; 18.64%).

Accidental MDs are of short duration and are usually associated with closely related populations. In the present systematic review, out of 20 MDs, there were 12 accidental MDs: 7 air crashes [12–14,16,20,21,24] (23.80%), 2 ferry accidents [6,9] (9.52%), 2 train accidents [12,23] (9.52%) and 1 vehicle accident (4.76%) [22]. Such MDs may be associated with fewer victims, such as the Nepal air crash [14], the Barcelona train accident [23] and the Germany vehicle accident [22] (n = 18, n = 12 and n = 8 victims, respectively). However, disasters such as the Estonia ferry disaster [11] (n = 852), the Croatia air crash [12] (n = 176), the Croatia train accident [12] (n = 152) and the Nigeria air crash [24] (n = 152) were associated with a large number of victims. In these types of MDs, the forensic experts already know the list of individuals who are on board; hence, the retrieval of ante-mortem records is easily carried out. The easy availability of ante-mortem records for all the victims is responsible for the higher percentage of victim identification in these MDs. This is reflected in the Barcelona train accident [23], the Newark air crash [21] and the Germany vehicle accident [22], where all the victims were identified. Apart from these MDs, the largest number of victims was identified in the France air crash [13] (n = 177; 97.7%), followed by the Nigeria air crash [24] (n = 148; 97.36%), the Croatia air crash [12] (n = 166; 94.31%) and the Kentucky air crash [20] (n = 47; 94%). In contrast, lower identification rates were observed in the Indonesia air crash [16] (n = 6; 5.7%) and the Europe ferry disaster [11] (n = 94; 11.03%). Disasters associated with industries and the military can cause difficulty in victim identification due to similar ages, sex, ethnic backgrounds and clothing (uniforms) of the victims. However, the related literature on industrial and military MDs was not available for analysis at this time.

A literature search revealed three criminal MDs [9,10,17]. Unlike natural and accidental MDs, criminal MDs may occur over extremely long periods of time and across wide ranges of territory. The remains of the victims of serial killers may be hidden, dismembered and mutilated. Dental structures in these situations may not be available for post-mortem review. The bodies of victims are sometimes mutilated to such an extent that only DNA identification can be used. In the Croatia criminal MDs [9,10], authors reported the problems encountered during identification of victims recovered from four mass graves [9]. Supportive and anthropological findings were the most common modes of identification (43%), followed by dental records (16%). Dental prosthesis records (fixed and removable prosthetic appliances) were the most common records used for the identification.

EM-DAT is a global database of natural and technological disasters containing essential core data on the occurrence and effects of more than 21,000 disasters worldwide, from 1900 to the present. Preliminary EM-DAT data collected in 2017 showed that 149 disasters occurred in 73 countries and resulted in 3,162 deaths. This finding emphasizes the volume of MDs occurring all over the world [26]. Intriguingly, we found only 19 papers (20 MDs) in the literature, which indicates the dire need to report victim identification in future MDs to better understand the role of forensic experts and odontologists. This reporting will help us to develop and streamline standard procedures for victim identification in MDs.

Of the 20569 identified victims (86.95%) in the present review, 16447 victims (79.96%) were identified using methodologies other than FO. Among these, visual identification [8,11,14,15,24] [14029 (68.2%)] and fingerprinting [10,11,14–16,24] [1250 (6.07%)] are simplest and most reliable methods of identification. DNA fingerprinting was also employed as a measure of victim identification alone (n = 241; 1.17%) or together with other means of fingerprinting + DNA methods (n = 9; 0.04%), fingerprinting + physical methods + DNA methods (n = 12; 0.06%), and physical methods + DNA methods (n = 384; 1.85%) [14,16,18,21,22,24]. Although DNA fingerprinting is the most reliable and accurate method for victim identification, it is a costly, technique-sensitive and time-consuming procedure and requires sophisticated machines and fully trained lab specialists. It also requires that the samples be untainted, as tainted samples are useless for testing, which could be a major limitation for the use of this technique in MDs. A small amount of human error (such as exposing the sample to other substances or incorrectly identifying two samples as identical) can ruin the process or alter the results.

A total of 1094 victims (5.31%) from seven MDs were identified using FO in combination with other modalities [10,12,14,15,19,24]. In such identifications, physical examination [14,15] (n = 299), DNA analysis [15,19,24] (n = 201) and fingerprinting [15,19] (n = 143) were the most commonly used modalities. Details about other methodologies used along with FO were not available for the Croatia criminal incident [10], train accident [12] and air crash [12]. These data suggest that forensic odontological means can be a good adjunctive method for victim identification.

Of the 20569 victims (86.95%) identified, FO was useful in the identification of 3025 victims (14.70%). The ante-mortem and post-mortem dental factors that can be used for comparison are teeth (number, type, position, crown and root morphology, crown and root pathology, pulp chamber, root canal morphology, etc.), dental prostheses (restorations, fixed and removable prosthesis, implants), periodontal ligaments, jawbones and pathologies associated with jawbones. Most dental identifications are based on comparisons of restorations, caries, missing teeth and/or prosthetic devices, leading to easy documentation in the records [27]. All these records can be obtained from general dental practitioners (GDPs) or teaching dental hospitals as ante-mortem data. Thus, we believe that the preservation of dental records by GDPs and/or dental hospitals is of paramount importance. Each GDP has a responsibility to understand the forensic implications associated with the practice of his or her profession. The production, retention, and release of clear and accurate patient records are essential parts of the dentist’s professional responsibility. Unfortunately, in India, there is inadequate knowledge and a lack of practice regarding proper record maintenance among GDPs [28]. A dire need has been proposed in many studies for creating awareness among the practitioners regarding proper dental record maintenance and its future use in victim identification in MDs.

The quality of ante-mortem data is the sole regulatory parameter for effective victim identification in FO. This is quite evident for the Thailand tsunami MD wherein due to the poor availability of ante-mortem dental data, only a small number of Thai victims could be identified using FO. In contrast, the dental identification rate for foreign victims was approximately 80% [29,30]. However, in the Japan tsunami, odontologists had difficulties in obtaining ante-mortem dental records of the victims because many dental offices were destroyed by the tsunami [25].

## Conclusion

FO has played a significant role in victim identification in some of the MDs across the world. It has also been used in combination with other methodologies for successful victim identification, thus making it a good adjuvant method. FO is considered to be one of the most reliable and economical scientific methods for MD management. However, the success of FO-mediated identification is heavily dependent on the availability of ante-mortem records from GDPs. Hence, adequate knowledge about FO and appropriate dental record keeping among GDPs are critical. With an increasing population, changing climatic conditions, faster public transportation avenues and increasing criminal activities, the incidence of MDs will likely increase in the future. Hence, GDPs should realize their national responsibilities in such grave situations.

## Supporting information

S1 Table PRISMA checklist. (DOC)
